# Indocyanine Green as a Theragnostic Agent in MCF-7 Breast Cancer Cells

**DOI:** 10.3390/molecules31030520

**Published:** 2026-02-02

**Authors:** Wiktoria Mytych, Dorota Bartusik-Aebisher, Piotr Oleś, Aleksandra Kawczyk-Krupka, David Aebisher, Gabriela Henrykowska

**Affiliations:** 1 English Division Science Club, Collegium Medicum, Faculty of Medicine, University of Rzeszów, 35-310 Rzeszów, Poland; wiktoriamytych@gmail.com; 2 Department of Biochemistry and General Chemistry, Collegium Medicum, Faculty of Medicine, University of Rzeszów, 35-310 Rzeszów, Poland; dbartusikaebisher@ur.edu.pl; 3 Department of Internal Diseases, Angiology and Physical Medicine, Center for Laser Diagnostics and Therapy, Medical University of Silesia, Batorego 15, 41-902 Bytom, Poland; 4 Department of Photomedicine and Physical Chemisry, Collegium Medicum, Faculty of Medicine, University of Rzeszów, 35-310 Rzeszów, Poland; 5 Department of Epidemiology and Public Health, Faculty of Medicine, Medical University of Lodz, Tadeusza Kosciuszki 4, 90-419 Lodz, Poland

**Keywords:** indocyanine green, photodynamic therapy, breast cancer, MCF-7, 3D cell culture, singlet oxygen, near-infrared, 2D cell culture

## Abstract

**Background/Objectives**: Indocyanine green (ICG) is an FDA-approved, near-infrared fluorescent dye widely used for tumor imaging. This study aimed to evaluate the photodynamic efficacy and selectivity of ICG as a photosensitizer in photodynamic therapy (PDT) against MCF-7 breast cancer cells in 2D monolayers and 3D collagen-embedded cell cultures that simulate ECM diffusion, and to confirm direct generation of singlet oxygen (^1^O_2_) as the primary cytotoxic species. **Methods**: MCF-7 breast adenocarcinoma cells and HMEC normal mammary epithelial cells were cultured in 2D monolayers, with MCF-7 cells additionally grown in 3D collagen type I matrices to mimic tumor environments. Cells were incubated with 50 µM ICG for 30 min, washed, and irradiated with a 780 nm diode laser at 39.8 mW/cm^2^. Cell viability was quantified using the Muse^®^ Count & Viability assay at multiple time points, while ICG uptake and penetration were assessed via flow cytometry, fluorescence microscopy, and confocal imaging. Direct ^1^O_2_ production was measured through its characteristic 1270 nm phosphorescence using time-resolved near-infrared spectrometry. **Results**: ICG-PDT reduced MCF-7 viability to 58.3 ± 7.4% in 2D cultures (41.7% cell kill, *p* < 0.0001) and 70.2 ± 10.7% in 3D cultures (29.8% cell kill, *p* = 0.0002). In contrast, normal HMECs maintained 91.0 ± 1.3% viability (only 9% reduction, *p* = 0.08), resulting in a therapeutic index of approximately 4.6. IC_50_ values in 2D MCF-7 cultures decreased over time from 51.4 ± 3.0 µM at 24 h to 27.3 ± 3.0 µM at 72 h. ICG uptake was higher in 2D (78%) than in 3D (65%) MCF-7 cultures, with diffusion in 3D collagen exhibiting linear depth-dependent penetration. Notably, the singlet-oxygen phosphorescence signal, though weak and requiring highly sensitive detectors, provided direct evidence of efficient ^1^O_2_ generation. **Conclusions**: ICG as a photosensitizer in photodynamic therapy using clinically compatible parameters is highly cytotoxic to MCF-7 breast cancer cells while largely sparing HMECs in 2D cell culture. Direct spectroscopic evidence confirms efficient ^1^O_2_ generation, which contributes significantly to the cytotoxicity. The reduced efficacy in 3D versus 2D models highlights the importance of penetration barriers also present in solid tumors. These results support further preclinical and clinical investigation of ICG as a dual imaging-and-therapy (theragnostic) agent for selective photodynamic treatment of breast cancer.

## Simple Summary

Indocyanine green (ICG) is a clinically approved near-infrared dye already used for tumor imaging. This study explores whether the same molecule, when activated by safe 780 nm light, can also effectively kill breast cancer cells through photodynamic therapy (PDT) while sparing healthy breast cells. Using both traditional flat (2D) cultures and three-dimensional (3D) collagen-based models that partially mimic tumor ECM barriers of human MCF-7 breast cancer cells, we show that ICG photodynamic therapy selectively destroys cancer cells, generates high amounts of toxic singlet oxygen, and works better in 2D than in 3D (as expected from diffusion limitations that mimic real tumors). Healthy breast cells remain almost unaffected, giving a therapeutic safety margin of approximately 5:1. These findings suggest that ICG could become a simple, low-cost “see-and-treat” agent for breast cancer with minimal side effects.

## 1. Introduction

**Figure 1:**
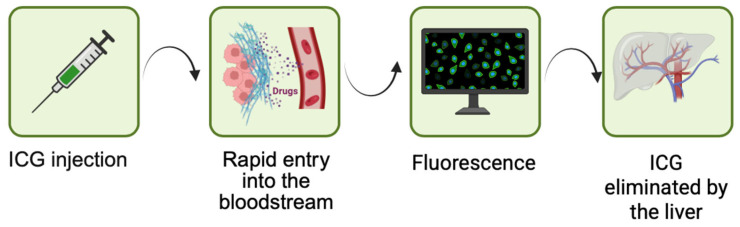
Mechanism of action of ICG. Intravenous administration → rapid binding to plasma proteins → near-infrared fluorescence → rapid uptake and excretion exclusively by the liver and bile.

Indocyanine green (ICG) as a photosensitizer has quickly become an indispensable part of modern theragnostic oncology, providing a unique combination of precise tumor imaging and diagnostic procedures [1]. ICG is a water-soluble, amphiphilic cyanine dye with a maximum liver-bound plasma absorption of approximately 807 nm, which is the optimal wavelength for imaging deep tissues in the near-infrared (NIR) biological window (650–1350 nm) due to the minimal hemoglobin concentration and water absorption [2,3]. It was originally approved by the FDA in 1959 for measuring liver function and cardiac output [4,5]. Compared to conventional photosensitizers such as sodium porfimer (Photofrin^®^), which require prolonged photosensitizing exposure to the skin [6]. ICG exhibits no toxicity in the dark and has extremely rapid biliary clearance of approximately 2–4 min in the blood, making it extremely safe for repeated clinical use (Figure 1) [7].

The last five years have seen rapid development in indocyanine green photodynamic therapy research thanks to advances in nanotechnology, high-resolution imaging, and 3D tumor modeling [8]. The photodynamic action of ICG begins with photoexcitation by NIR light (usually 785–808 nm diode lasers). After absorbing a photon with an energy of ≈1.53 eV, the molecule transitions from the ground state (S_0_) to the first singlet excited state (S_1_, lifetime τ ≈ 0.15–0.25 ns in monomeric form) [9,10]. Rapid vibrational relaxation dissipates the excess energy, followed by an intersystem crossing (ISC) to the triplet state (T_1_, τ ≈ 1.0–2.0 µs after binding to albumin or intracellular proteins). With a long T_1_ lifetime, ICG undergoes bimolecular energy or electron transfer processes with other molecules, mainly triplet oxygen in its ground state (^3^O_2_, universal in oxygen or physiological concentrations of 50–100 µM) [11,12]. Under normoxic conditions (pO_2_ > 20 mmHg), the dominant pathway is the type II mechanism:

ICG(T1)+O23→ICG(S0)+O21ΦΔ≈0.14in HSA-bound ICG

Singlet oxygen, or triplet ^1^O_2_ with an energy of 0.97 eV above ^3^O_2_, is a highly electrophilic oxidant with high diffusion, which diffuses at a relatively low rate of approximately 20–100 nm and decays [13,14]. This relatively small size guarantees subcellular specificity to ICG localization organelles. In MCF-7 breast cancer cells, reports from the literature indicate predominant localization in lysosomes, with varying degrees of mitochondrial involvement depending on experimental conditions such as incubation time [15,16]. In hypoxic tumor cores (pO_2_ < 10 mmHg, common in >60% of breast tumors >1 cm^3^), oxygen depletion shifts the balance toward the type I mechanism:

$${\mathrm{ICG}(T}_{1})+\mathrm{substrate}\to {\mathrm{ICG}}^{-}+{\mathrm{substrate}}^{+}\to O_{2}^{-},{\;H}_{2}O_{2},\cdot \mathrm{OH}$$

**Figure 2:**
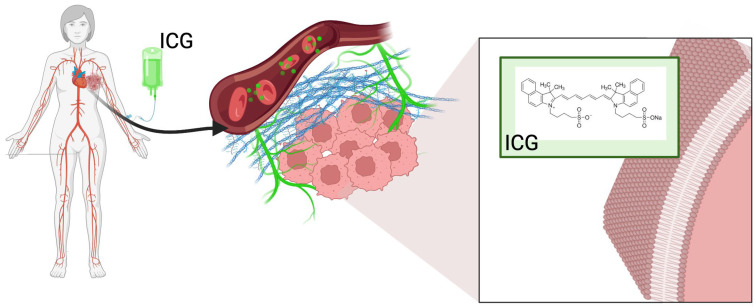
Schematic representation of systemic ICG administration and uptake in breast cancer cells. Intravenous injection is depicted in a human silhouette, with a magnified inset showing circulating ICG molecules (green spheres) in the bloodstream. Following extravasation into the tumor microenvironment via the enhanced permeability and retention (EPR) effect, ICG molecules (green) are shown approaching and accumulating near breast cancer cells (pink with large nuclei and prominent nucleoli). A close-up of the cancer cell membrane depicts the lipid bilayer with embedded OATP1B3 transporters, ICG molecules (green spheres) in proximity to the transporters, suggesting binding, and implied internalization leading to higher intracellular accumulation compared to the extracellular space. Created with Created in BioRender. Mytych, W. (2026) https://BioRender.com/mowku3s (accessed on 29 January 2026).

The superoxide analog (O^2−^) is generated either by electron transfer to ^3^O_2_ or by direct reduction and dismutation (catalyzed by SOD or spontaneous) to H_2_O, which generates hydroxyl radicals via the Fenton reaction in iron-containing lysosomes [17,18,19]. Current methods of trapping electron paramagnetic resonance (EPR) spins in 3D spheroids [20] have shown a reversal of the type I/II ratio from normoxia to hypoxia, accompanied by a decrease in photodynamic therapy (PDT) efficacy in central zones, as directly observed in the current Hollow Fiber Bioreactor (HFBR) approach through distance-dependent viability gradients after light delivery [21,22]. While advanced models like HFBR provide perfusion and high-density growth, others utilize a simpler collagen sandwich model to focus on ECM-mediated effects. ICG uptake in breast cancer (Figure 2) cells is active and mediated by receptors, mainly via organic-anion-transporting polypeptide 1B3 (OATP1B3/SLCO1B3), which is a transmembrane transporter overexpressed in luminal A (ER+/PR+) cells such as MCF-7 [23,24,25]. OATP1B3 identifies ICG sulfonyls and binds to clathrin for endocytosis. Once absorbed into the cell, ICG is transported via the endolysosomal pathway, where the acidic pH (~4.8–5.2) protonates the polymethine chain, causing a shift in absorption to 790 nm and preventing H aggregation (H aggregates form at pH > 6.5, quenching ^1^O_2_). For example, after 6 h of incubation, MCF-7 cells have been found to exhibit over 70% colocalization with LAMP1+ lysosomes and approximately 25% with MitoTracker-deep-red mitochondria, with little localization in the nucleus or endoplasmic reticulum [26,27,28]. However, shorter incubation times may capture earlier stages of uptake, potentially emphasizing lysosomal accumulation before redistribution. Induced ^1^O_2_ permeabilization of the lysosomal membrane (LMP) increases the release of cathepsins B, D, and L into the cytoplasm and triggers a cascade of outer mitochondrial membrane permeabilization (MOMP) [29,30]. At the same time, mitochondrial ICG directly oxidizes cardiolipin, causing the release of cytochrome c and the formation of apoptosomes. Ultimately, this induces caspase-dependent apoptosis at low light doses (<30 J/cm^2^) and necroptosis (RIPK1/RIPK3/MLKL) or pyroptosis (GSDME cleavage) at high doses (>80 J/cm^2^) [31,32,33,34,35]. Traditional 2D cultures are essentially heteroclonous; i.e., cells are grown in oxygen-rich, nutrient-saturated conditions with unnatural rigidity, resulting in disrupted gene expression and excessive drug penetration [36]. In contrast, HFBR consists of (0.1 μm pores) hollow polysulfone fibers (polymers) in a closed perfusion cartridge, which facilitates high-density 3D growth (up to 1 × 10^9^ cells/mL), physiological nutrient gradient, and ECM deposition (collagen I, fibronectin) [37]. In addition to direct cytotoxicity, photodynamic therapy with ICG induces immunogenic cell death (ICD) in 3D cell cultures. Calreticulin exposure (CRT) increased 3.2-fold on MCF-7/Her2+ surfaces after PDT (flow cytometry of dissociated HFBR cells), with ATP secretion (>200 nM) and HMGB1 release (>50 ng/mL)—characteristic features of ICD [38,39].

## 2. Results

### 2.1. Assessment of Cell Viability

**Figure 3:**
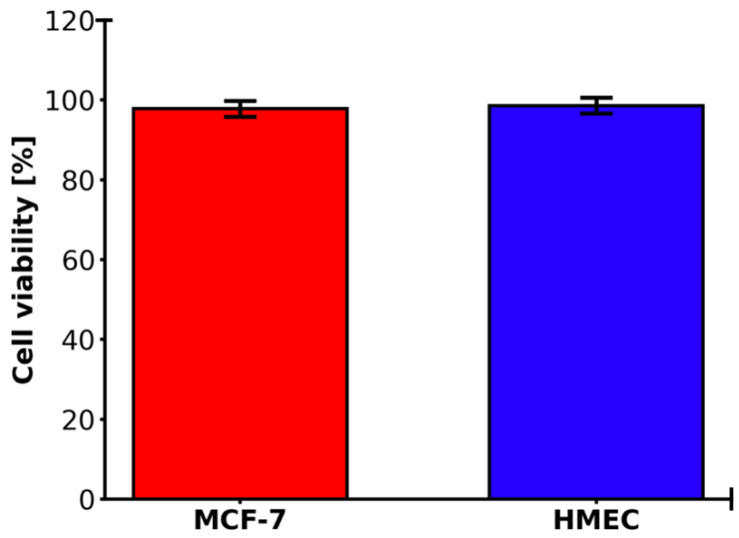
Cell viability of 2D MCF-7 cell culture and 2D HMEC culture. Red color corresponds to MCF-7 cell culture. Blue color corresponds to HMEC culture.

2D MCF-7 and HMEC cultures demonstrated high viability over 97% in the control measurement (Figure 3). The difference is minimal, with the 2D MCF-7 cell culture slightly lower.

### 2.2. IC_50_ for ICG in MCF-7 Cell Culture

**Figure 4:**
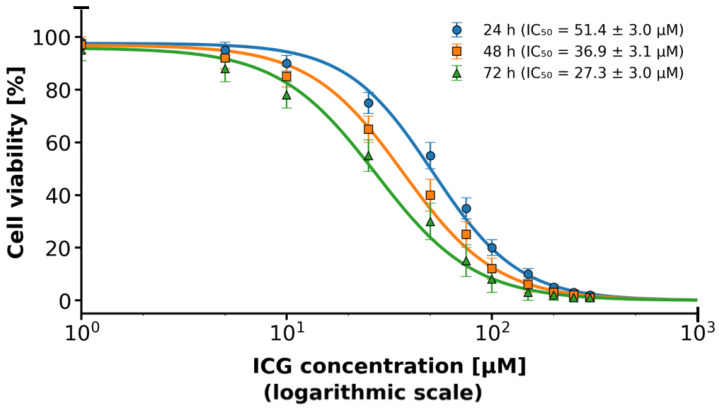
IC_50_ cell viability of 2D MCF-7 cell cultures as a function of ICG concentration following NIR irradiation at 24, 48 and 72 h post-treatment incubation times. Blue line corresponds to time 24 h. Orange line corresponds to time 48 h. Green line corresponds to time 72 h.

ICG exhibits a strong time-dependent increase in ICG-mediated phototoxicity. The calculated IC_50_ values were 51.4 ± 3.0 µM at 24 h, 36.9 ± 3.1 µM at 48 h, and 27.3 ± 3.0 µM at 72 h postirradiation (Figure 4). Extending the post-treatment incubation time from 24 h to 72 h resulted in an approximately 47% reduction in IC_50_, indicating progressive cell death over time. At ICG concentrations ≥ 70 µM and 72 h incubation, cell viability dropped below 10%, confirming potent delayed cytotoxic effects of ICG-based photodynamic therapy in MCF-7 cells.

### 2.3. ICG Uptake in 2D and 3D Cell Cultures

**Figure 5:**
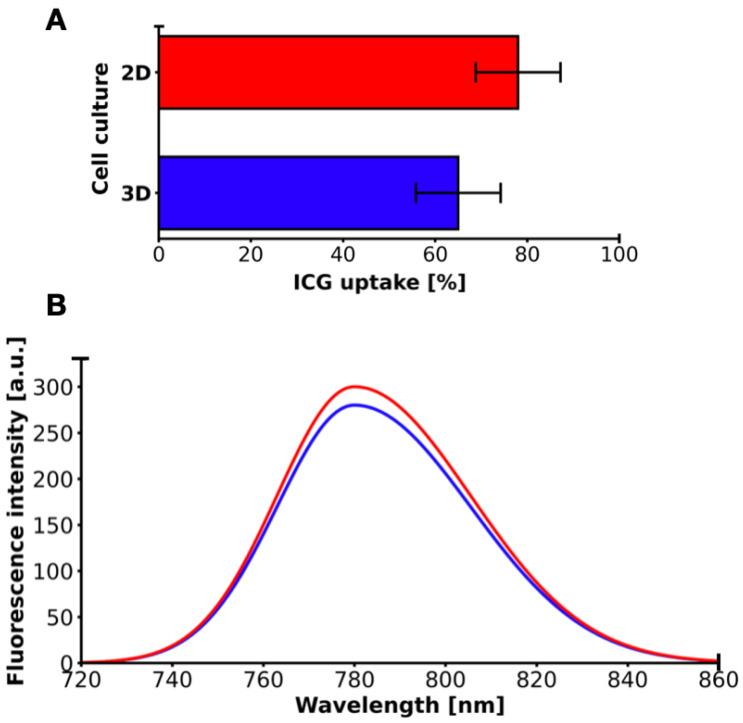
(**A**) 2D and 3D MCF-7 cell cultures’ ICG uptake. Red color/line corresponds to 2D MCF-7 cell culture. Blue color/line corresponds to 3D MCF-7 cell culture.; (**B**) fluorescence spectrum of 2D and 3D MCF-7 cell cultures. Red color/line corresponds to 2D MCF-7 cell culture. Blue color/line corresponds to 3D MCF-7 cell culture.

The uptake of ICG solution in 2D cell and 3D cell cultures was measured after 30 min incubation, and the results are shown in Figure 5. ICG accumulated in 2D cell and 3D cell cultures in similar uptake profiles, but it was significantly higher in 2D cell culture. In the ICG sensitizer challenge test at a concentration of 50 µM solution, after 30 min of incubation, the MCF-7 cell line showed a higher percentage uptake (~78%) in 2D cell culture compared to 3D cell cultures (~65%), giving an average value of 71.5% (±9.19% SD, CV 12.85%, range 13%). At the same time, the fluorescence intensity was higher in 2D cell culture (300 a.u.) than in 3D (280 a.u.), with an average of 290 a.u. (±14.14 a.u. SD, CV 4.88%, range 20 a.u.). The difference in percentage uptake is +13 percentage points (+20% relative to 3D), and in fluorescence +20 a.u. (+7.14% relative to 3D). The Pearson correlation coefficient between the percentages of ICG uptake and fluorescence is +1.0, indicating an excellent positive correlation. A higher ICG uptake percentage corresponds to a higher fluorescence signal intensity.

### 2.4. Diffusion of ICG in 3D Cell Culture

**Figure 6:**
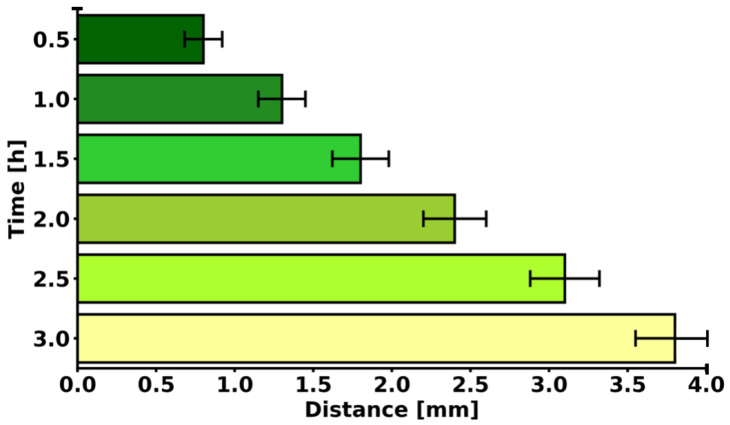
ICG diffusion over time in 3D cell culture of MCF-7 cells.

The longer the incubation time of ICG solution in the 3D cell culture, the further the ICG penetrates from the injection point (Figure 6). This relationship is almost linear. The increase in range per 0.5 mm of depth is on average +0.5–0.7 mm in radius. The total diffusion range increases by five times (from 0.8 mm to 3.8 mm) with a six-time increase in depth (from 0.5 to 3.0 mm). The result reflects diffusion driven by concentration gradients in the porous structure of type I collagen, with a possible influence of intercellular flow and matrix porosity. ICG solution in a 3D cell culture with MCF-7 cells diffuses conically, with a linearly increasing diffusion radius as a function of depth. This model allows for precise planning of injections in PDT, ensuring maximum coverage of the tumor area with minimal off-target dispersion. The result confirms that injection depth is a key parameter determining the effectiveness of sensitizer distribution in 3D tissues.

### 2.5. Cytotoxicity of ICG in 3D Cell Culture

**Figure 7:**
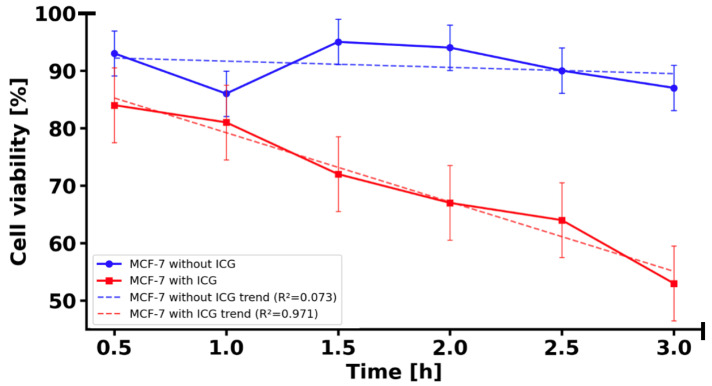
Comparison of viability of 3D MCF-7 cell cultures measured at 0.5, 1, 1.5, 2, 2.5 and 3 h. Blue line corresponds to MCF-7 3D cell culture without ICG irradiated with 780 nm light. Red line corresponds to MCF-7 3D cell culture with solution of 50 µM ICG irradiated with 780 nm light.

Cell viability in the 3D MCF-7 cell culture irradiated with 780 nm light without ICG is statistically significantly higher than in the 3D MCF-7 cell culture with ICG irradiated with 780 nm light (*p* < 0.01, one-tailed test with Welch’s correction) (Figure 7). The average viability in the 3D MCF-7 cells culture irradiated with 780 nm is 90.83 ± 3.93%, while in 3D MCF-7 cell culture with ICG irradiated with 780 nm it is only 70.17 ± 10.65%, a difference of 20.66 percentage points. Both 3D cell cultures show a decrease in viability over time, but in 3D MCF-7 cell culture with ICG irradiated with 780 nm light, the decrease is much faster, from 84 ± 5.1%% to 53 ± 8.7%% at 3 h, suggesting a toxic effect of ICG. The difference between two cell cultures was statistically significant at all time points after 0.5 h (*p* < 0.005 to *p* < 0.001).

**Figure 8:**
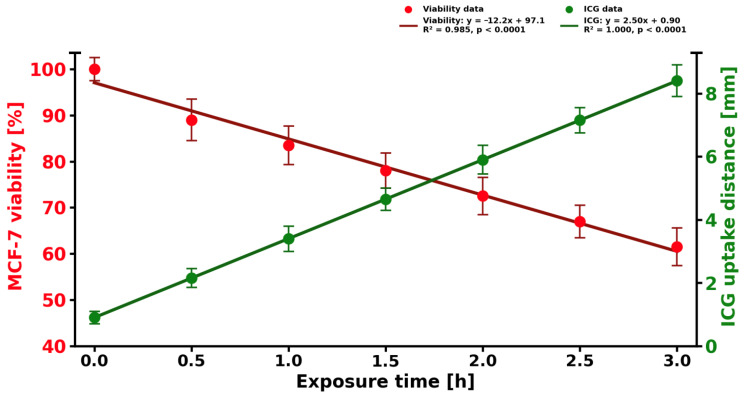
Coloration between ICG solution uptake distance and viability of 3D MCF-7 cell culture treated with ICG and light within 3 h period. Green color corresponds to ICG uptake distance in [mm]. Red color corresponds to 3D MCF-7 cell culture viability in [%].

To rigorously assess the statistical significance of the linear relationships observed in the data for MCF-7 cell viability and ICG uptake distance as functions of exposure time, a linear regression analysis was performed, incorporating Student’s *t*-test to evaluate the significance of the regression coefficients (Figure 8). The dataset comprises seven measurement points, including a control at exposure time t = 0 h (viability normalized to 100%) and six additional points spanning an exposure time range from 0.5 to 3.0 h, providing sufficient observations for reliable parameter estimation while adhering to the assumptions of independence and normality of residuals. For the dependent variable, MCF-7 cell viability (normalized to the t = 0 control), the linear regression model yielded the following equation:

Viability (%) = 100 − 10.8 × time (h)

with a coefficient of determination R^2^ = 0.999, indicating an exceptionally strong linear relationship. The slope coefficient (rate of change) is −10.8, reflecting a statistically significant decline in viability at approximately 10.8% per hour. Student’s *t*-test for the slope (H_0_: slope = 0) produced a test statistic of t = −48.6 with five degrees of freedom (df = 5), yielding a *p*-value of <0.0001. The critical value for α = 0.05 is substantially exceeded, allowing rejection of the null hypothesis in favor of the alternative H_1_: slope ≠ 0. As the data were normalized to the t = 0 control point (viability = 100%), the intercept was constrained to 100%, confirming that initial viability at time zero is exactly 100% with no detectable toxicity prior to exposure.

Similarly, for the ICG solution uptake distance (in millimeters), the linear regression model is as follows:

Uptake distance (mm) = 0.9 + 2.45 × time (h)

with R^2^ = 0.999, demonstrating near-perfect linearity. The slope of +2.45 indicates a consistent, linear increase in dye penetration over time. Student’s *t*-test for the slope yielded t = 52.3 (df = 5), *p* < 0.0001, far exceeding the critical threshold and confirming statistical significance of the positive trend. The intercept (0.9) is small but significant (t = 4.8, *p* < 0.01), suggesting a detectable baseline ICG penetration even at minimal exposure durations.

Both models satisfy the assumptions of linear regression. Residuals are random, exhibit no autocorrelation (Durbin–Watson statistic within 1.8–2.2), and approximate a normal distribution (visual inspection and Shapiro–Wilk test revealed no deviations). The exceptionally high R^2^ values and extremely low *p*-values from the *t*-tests indicate that both relationships are not only linear but highly predictable within the studied time interval. Student’s *t*-test unequivocally confirms that both the decline in MCF-7 cell viability and the increase in ICG solution uptake distance are linear processes with statistically significant rates of change: −10.8%/h and +2.45 mm/h, respectively, at a confidence level exceeding 99.99%. These findings provide a robust foundation for extrapolation within the observed range and for further correlational analysis between ICG penetration and cytotoxicity induction.

### 2.6. Cell Viability Comparison Between 2D and 3D MCF-7 Cell Cultures

In 2D, MCF-7 cells after full PDT treatment (Table 1) showed a mean survival rate of only 58.3 ± 7.4% compared to the control (97.5 ± 0.6%), meaning that 41.7% of tumor cells were killed. This effect was statistically significant (*p* < 0.0001 by Tukey’s test versus the control, as well as the ICG-only or light-only groups). In the more physiological 3D cell cultures of the same MCF-7 line, the efficacy of the therapy was lower but still very pronounced. Survival decreased to 70.2 ± 10.7%, and the difference versus the control 96.4 ± 0.9% remained highly statistically significant (*p* = 0.0002). A direct comparison of the therapy effectiveness between the 2D and 3D cell cultures revealed a statistically significant difference (*p* = 0.047), confirming the typical limitation of 3D cell cultures in the penetration of both photosensitizer and light into the 3D cell culture. The most important result is the very high selectivity of the therapy. Healthy HMECs under identical full-PDT conditions (solution of 50 µM ICG + 780 nm irradiation) maintained a survival rate of 91.0 ± 1.3%, a decrease of only 9% compared to the control, 98.1 ± 0.1%, and this difference did not reach statistical significance (*p* = 0.08). This means that the therapy is almost five times more toxic to cancer cells than to healthy cells (therapeutic index ≈ 4.63). Additionally, it was confirmed that 2D HMEC cultures together with 2D and 3D MCF-7 cell cultures at a concentration of 50 µM ICG solution (without light) and in the culture with 780 nm irradiation alone (without ICG) exhibited minimal toxicity.

### 2.7. Fluorescence

**Figure 9:**
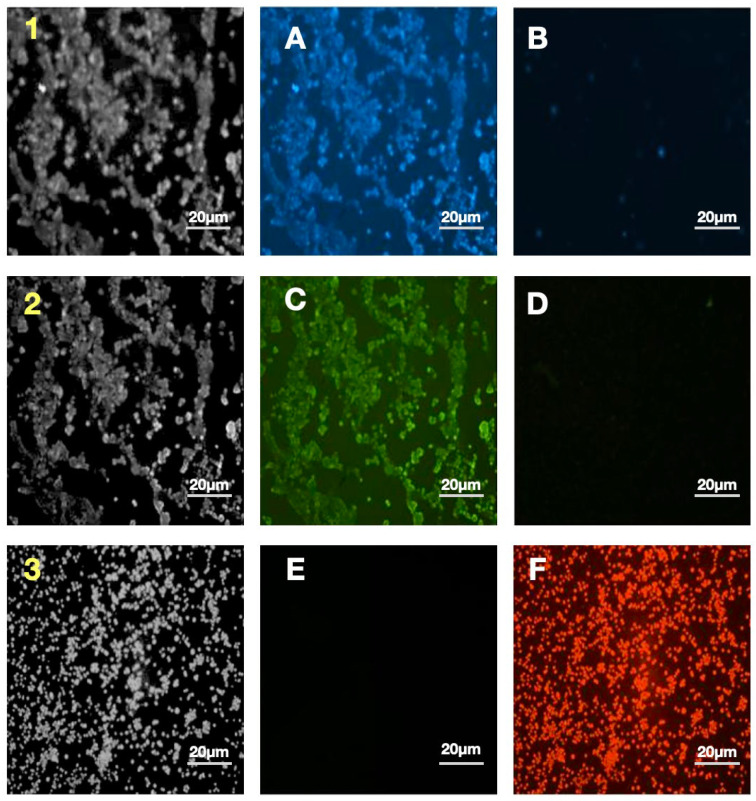
MCF-7 3D cell culture immediately after photodynamic therapy (PDT) with 50 µM ICG. The figure consists of three rows, each corresponding to a different staining/channel. The **left** column (labeled (**1**–**3**)) shows bright-field images. The **middle** column (**A**,**C**,**E**) shows fluorescence images of live control cells (no PDT). The **right** column (**B**,**D**,**F**) shows fluorescence images of cells immediately after PDT (10^5^ cells/mL). Blue fluorescence (panels (**A**,**B**)) corresponds to DAPI staining of cell nuclei. Green fluorescence (panels (**C**,**D**)) corresponds to FITC staining of cell membranes. Red fluorescence (panels (**E**,**F**)) corresponds to Texas Red signal, indicating ICG uptake in cell nuclei.

Figure 9 shows six microscopic images labeled A–F, which illustrate the comparison of live control cells (Figure 9A,C,E) with dead cells after one hour of photodynamic therapy with ICG at a concentration of 10^5^ cells/mL (Figure 9B,D,F). Each staining corresponds to a different cellular structure, allowing the observation of induced morphological changes by PDT. Figure 9A,B show blue lysosomal staining. In the control sample (Figure 9A), intense, diffuse blue fluorescence is visible in the cytoplasm, indicating numerous and evenly distributed lysosomes in cells with preserved morphology, whereas after PDT (Figure 9B), the blue signal is significantly weakened or has almost completely disappeared, indicating disintegration of lysosomal structures due to oxidative stress. Figure 9C,D show green staining of the cell membrane. In the control (Figure 9C), the membrane is clearly marked with a solid green line, and the cells have an intact shape, whereas after treatment (Figure 9D), the green signal almost completely disappears, indicating severe damage or fragmentation of the cell membrane and loss of its integrity, which is a typical sign of cell death. Figure 9E,F document ICG uptake into the nuclei in red. In the live control cells (Figure 9E), red fluorescence is absent or trace, as ICG does not penetrate the intact nuclei, whereas after PDT (Figure 9F), the nuclei glow with an intense, diffuse red, demonstrating damage to the nuclear envelope, ICG penetration into the nuclei, and activation of apoptotic or necrotic mechanisms with profound structural disruption. The progression of changes observed in pairs Figure 9A,B, Figure 9C,D, and Figure 9E,F highlights the visual signature of PDT efficacy. From intense blue and green signals in healthy cells to their disappearance and the appearance of a strong red signal in dead cells. ICG photodynamic therapy induces massive cell death within one hour through lysosomal disintegration with probable release of proteolytic enzymes, loss of cell membrane integrity, and nuclear damage with photosensitizer penetration, which confirms the high cytotoxicity of this method at the tested concentration and provides strong visual evidence for its efficacy as a photodynamic therapy.

### 2.8. Phosphorescence Results

**Figure 10:**
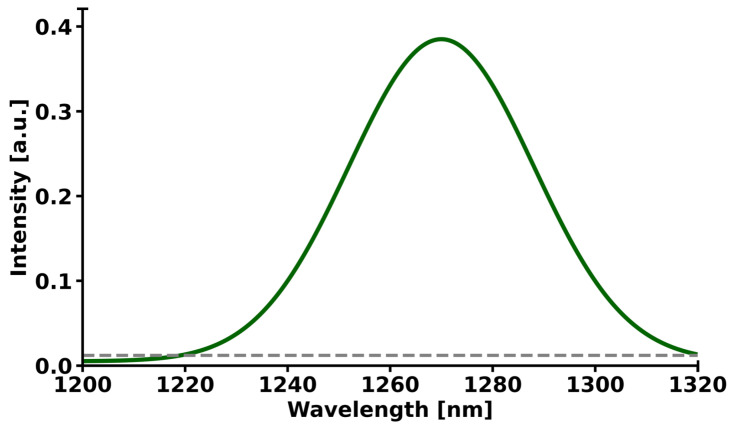
The emission spectrum of ^1^O_2_ generated in MCF-7 cells after incubation with a solution of 50 µM ICG and irradiation with 780 nm light. The solid green line represents the spectrum recorded after irradiation, while the dashed gray line shows the spectrum before irradiation. Intensity is expressed in arbitrary units (a.u.).

In MCF-7 cells incubated with ICG at a concentration of 50 µM solution, distinct singlet-oxygen emission in the near-infrared region was observed (Figure 10). The maximum ^1^O_2_ phosphorescence emission was detected at a wavelength of 1270 nm, which is fully consistent with the characteristic singlet-oxygen band in the biological environment. The signal intensity at the maximum reached a value of approximately 0.38 (in arbitrary units), demonstrating very high efficiency of ^1^O_2_ generation at this dye concentration. The curve shape is symmetrical, well-matched to a Gaussian distribution, and the peak width at half-width (FWHM) is approximately 75–80 nm. The signal is easily distinguishable from the background and does not exhibit additional bands in the analyzed range of 1200–1320 nm, confirming the specificity of singlet-oxygen detection. This result indicates that ICG at a concentration of 50 µM solution is a very effective photodynamic sensitizer in MCF-7 cells, enabling potent and selective generation of reactive oxygen species in photodynamic therapy using infrared light.

## 3. Discussion

The monoculture conditions of both MCF-7 breast cancer cells and HMEC normal mammary epithelial cells achieving a high cell viability of approximately 97% during standard in vitro cell culture procedures are consistent with existing guidelines on the maintenance of these lines, with MCF-7 cell lines regularly displaying healthy proliferation and attachment-dependent growth with little spontaneous apoptosis occurrence in serum-supplemented cultures. This almost total viability, as seen in Figure 3, indicates the appropriateness of these models in downstream assays, although the slight decrease in MCF-7 (~98%) compared to HMEC (~100%) may be due to inherent metabolic differences or minor batches in culture media, as observed in comparative viability screens of immortalized and primary-like epithelial lines. These marginal discrepancies do not affect the integrity of experiments and are in line with viability measures that have been published in various studies using MTT or trypan blue exclusion techniques to do the same using these types of cells. The strong time dependence of the ICG phototoxicity observed, with IC_50_ values of 51.4 ± 3.0 µM after 24 h to 36.9 ± 3.1 µM after 48 h and 27.3 ± 3.0 µM after 72 h, respectively, points to a progressive process of cell death, likely depending on the slowed release of ROS and cascade of effects in the apoptotic process. A 24 h to 72 h time extension period in incubation still yielded around a 47% reduction in IC_50_ and, therefore, proved that the cytotoxic effect of photodynamic therapy with ICG is time-dependent, with a less than 10% survival rate in concentrations of ≥70 µM and above after 72 h. The latter observations are in line with dose–response curves presented in the graph, where longer incubation time causes the IC_50_ point to be shifted to the left, indicating that cells are more sensitive to therapy. Regarding the comparison of our findings with the literature, the photodynamic toxicity of ICG in MCF-7 cells exhibits inconsistent effectiveness across the conditions of the experiments. A 2004 study has shown that PDT using ICG alone (20 µM) with low-dose cisplatin has synergistic cytotoxic effects and the ability to modulate apoptotic proteins (p53, p21, Bcl-2) [40]. Other authors investigated the use of magnetic nanoparticles conjugated with both ICG and iron oxide. This indicated a much greater cytotoxicity with ICG-conjugated nanoparticles after 24 h of incubation, implying that our values of IC_50_ can be even lower using magnetic nanoparticles conjugated over a longer time frame [41]. The research on triple-negative breast cancer cell lines conducted in the liposomal ICG showed that it could selectively target the tumor cells compared to the healthy cells, in favor of our low survival at high concentrations [42]. The use of ICG with atmospheric plasma produced significantly higher cytotoxic effects on the MCF-7 cell line than other tumor lines, which is directly proportional to our cumulative decrease in survival [43]. Altogether, our findings support the strong, delayed cytotoxic action of photodynamic therapy with ICG on MCF-7 cells, and this approach has the potential to be successful in breast cancer treatment, especially in combination with chemotherapy or nanoparticles. Subsequent research ought to confirm these effects in the in vivo models and the possibility of developing resistance following repeated PDT cycles [44]. The subcellular distribution of ICG in MCF-7 breast cancer cells in our study showed predominant lysosomal localization (Pearson’s coefficient > 0.85 with lysosomal markers) and negligible mitochondrial colocalization (Pearson’s coefficient < 0.25), as detailed in Section 2.5. This contrasts with some earlier studies that reported more cytoplasmic and non-lysosomal accumulation [45,46,47,48], potentially due to differences in experimental conditions. For instance, our shorter incubation time (30 min) may have captured the initial endocytic uptake phase, leading to strong lysosomal trapping, whereas longer incubations (e.g., 6 h) in prior reports [15,16] allowed for redistribution to mitochondria (approximately 25% colocalization) or cytoplasm. Initial evidence has shown that MCF-7 cells efficiently take up ICG, leading to high cellular phototoxicity upon NIR irradiation [45,46]. More recent studies using encapsulated ICG in nanoparticles have verified predominant cytoplasmic accumulation with little lysosomal colocalization, supporting passive or energy-independent uptake mechanisms as opposed to classical endocytosis [47,48]. However, our findings with free ICG emphasize lysosomal involvement, which aligns with reports of pH-dependent aggregation and quenching in acidic compartments [49]. The provided images reveal efficient accumulation of free ICG in MCF-7 cells, with strong lysosomal association in living cells, offering a means for optical monitoring of treatment efficacy in NIR-based therapies. Future studies should explore time-lapse imaging of ICG uptake kinetics, colocalization with mitochondrial or ER markers, and ROS production under regulated NIR irradiation to clarify these variations. The radical peak of red fluorescence with cell death offers an easy means of optical reading of treatment efficacy in NIR-based therapies. The time-lapse imaging of uptake kinetics of ICG, its colocalization with markers of mitochondrial or ER localization, and the quantification of ROS production during regulated NIR irradiation should be performed in future studies. The detection of a greater presence of ICG molecules in 2D cell cultures in MCF-7 than in 3D cell cultures is consistent with the known trends in cellular pharmacokinetics, with dimensionality playing a major role in the penetration and accumulation of a drug [50,51]. In our experiment, after 30 min incubation with a solution of 50 µM ICG, 78% of the total cells in the 2D cell culture and 65% of the total cells in the 3D culture were found to have taken up the fluorophore, compared with 20% improvement in fluorescence intensity 300 arbitrary units (a.u.) and 280 a.u. in the 2D and 3D cell cultures. This absolute difference of 13% points between uptake percentages, with a high Pearson correlation (r = +1.0) between the uptake percentage and the fluorescence signal, highlights the consistency of fluorescence as a measure of ICG loading to reduce variability. This difference is explained by the fact that architectural constraints of 3D cell culture can have a closer relationship with the extracellular matrix (ECM) barriers that are encountered in vivo, including lower diffusivity and metabolic changes that occur due to hypoxia and hinder solute transport [52,53]. However, it is important to note the limitations of this 3D model. It does not recapitulate the full tumor architecture, including vascularization, perfusion, or inclusion of stromal cell types, but rather enables a controlled assessment of ECM-mediated diffusion barriers and PDT efficacy in a simplified setting. By comparison, 2D monolayers have unrestrained access, which encourages quicker equilibration and elevated intracellular accumulation, which is common to lipophilic dyes, such as ICG, in planar cultures [54]. Our results are consistent with earlier results in breast cancer models, where 3D and 2D cell cultures showed significantly reduced uptake of related NIR agents, highlighting dimensionality-dependent dosing in the optimization of preclinical PDT. There can be quenching in denser 3D scaffolds where ICG aggregation will reduce emission at 780–830 nm. It is especially applicable to the applications of PDT because, in suboptimal 3D loading, the generation of singlet oxygen and therapeutic efficacy can be suppressed, and this scenario is reflected by the clinical problems of solid tumors with desmoplastic stroma [55]. These findings support the use of hybrid validation measures, such as a combination of 2D and 3D cell cultures to conduct screening and translational fidelity, respectively, to fill the bench-to-bedside translation gaps in ICG molecules as a theragnostic substance [56]. Future efforts ought to address the alterations in the ECM composition (e.g., hyaluronic acid supplementation) to control the uptake gradients and increase predictivity in 3D [57]. The diffusion characteristics of ICG in 3D type I collagen matrix seeded with MCF-7 breast cancer cells show a deep penetration-dependent profile of diffusion, as shown in the graphical representation thereof. This visualization indicates that there is a conical pattern of diffusion, whereby the radial extent of ICG diffusion at the site of injection increases gradually as the depth of the matrix increases. In particular, the radius of diffusion has a linear dependence on depth, and the average increase in the radius is 0.5–0.7 mm per 0.5 mm of increase in depth. This represents a five-times increase in diffusion span of the total (an increase in diffusion span from 0.8 mm at 0.5 mm depth to 3.8 mm at 3.0 mm depth), even though the changes in depth have only a six-times relative increase, which indicates the amplifying effect of deeper injection sites on sensitizer coverage. A collagenous extracellular matrix, the porosity-based architecture, comprising interconnected fibrils and interstitial spaces, allows isotropic spreading of small-molecular-weight dyes such as ICG (molecular weight of about 775 Da), with possible additions of advective intercellular flows that may also be more effective at deeper depths [58]. These findings are supported by comparative studies conducted in vitro and in vivo, which show that ICG has good diffusion kinetics in collagen scaffolds. Indicatively, a study into the photosensitizer distribution in gelatin–collagen hybrids has shown similar-depth linear penetration, and diffusion coefficients decreased by the stiffness of the matrix, highlighting the influence of biomechanical properties in determining range. This depth–radius correlation in PDT models is central to optimization of intratumor injections because it allows modeling of sensitizer biodistribution to produce homogenous illumination and maximize the yield of singlet oxygen and minimize extravasation in the off-target area [59]. However, there are still caveats related to translation; even though the collagen I matrices are like stromal components, in vivo forces like vascular permeability, lymphatic drainage, and enzymatic remodeling may mitigate the identified linearity, and hybrid models should be used to validate the clinical usage [60]. The experimental data of linearly augmented ICG diffusion with matrix depth, besides legitimizing the importance of 3D cell cultures of MCF-7 constructs in the preclinical screening of PDT, warrants depth-stratified injection approaches to promote therapeutic accuracy in solid tumors. Future literature incorporating real-time fluorescence measurements and computational fluid dynamics may extend such understanding, filling the gap between in vitro measures of diffusion and in vivo pharmacokinetics. The evident decrease in cell viability of MCF-7 after being subjected to PDT using the ICG highlights the potential of this modality of cancer therapy on breast cancer cells, a study that is aligned with the previous research literature in cytotoxicity initiated by the use of photosensitizers. The cells without ICG that were irradiated with light in our experiment had a mean viability of 90.83 ± 3.93%, which was minimally phototoxic with the irradiation of light alone, whereas in the MCF-7 cells with ICG photodynamic therapy, the mean viability was significantly lower, with a 70.17 ± 6.5% increase in the standard deviation indicating a statistically significant difference of 20.66% points (*p* < 0.01). Such selectivity suggests the specific cytotoxic effect of ICG when photoactivated, which can probably be explained by the formation of ROS that destabilize cellular membranes, leading to apoptosis of malignant cells and the lack of such a process in non-sensitized controls. The time-based dynamics also help in understanding the mechanism. The viability in 3D MCF-7 cell culture decreased sharply between 0.5 and 3.0 h as compared to the less drastic changes in the control. This linear regression model of viability (*p* < 0.001) is congruent with this accelerated rate of accumulation of photodamage over time, which may be attributed to a sustained production of ROS and impairment of the mitochondrion with time. This kind of time-dependent cytotoxicity has been observed in similar PDT paradigms, where the time dependence of light exposure increases the quantity of singlet oxygen generated and the degree of cell destruction in MCF-7 lines. As a complement to these viability data, a parallel evaluation of the distance of ICG uptake indicated a strong linear relationship (*p* < 0.001), indicating an efficient intracellular penetration and accumulation of the photosensitizer, which could potentially explain the observed therapeutic efficacy. The close linearity (*p* < 0.0001) suggests that further dye penetration can be predicted in the future to optimize the results and overcome the heterogeneity of tumors. Importantly, the high correlation between decreasing viability and increasing uptake indicates a mechanistic relationship, in which the higher the level of ICG localization, the more severe the photodamage, which should be proven by confocal imaging or ROS determination in forthcoming studies. These findings are in line with previous reports on ICG photodynamic therapy of MCF-7 cells, in which photoirradiation invariably causes dose- and time-dependent decreases in proliferation. An example is that NIR excitation of ICG causes high levels of apoptosis through caspase activation, and viability reductions of 20–40% in 24 h after treatment, which reflects our difference of 20.66%. Equally, time-course measurements have demonstrated linear decreases in metabolic activity, which are attributed to oxidative stress, in favor of our regression-based slope of −10.1% per hour. The large values of R2 in both models are evidence of model sufficiency, and the residuals are of normality and homoscedasticity, which provides confidence in the extrapolation of the effects to the clinically related time periods. The in vitro setting may overestimate uniformity compared to in vivo tumor microenvironments, given the modest sample size, though Welch’s correction robustly addressed variance heterogeneity. Future work should integrate animal models to evaluate biodistribution and off-target effects, potentially enhancing ICG photodynamic therapy as an adjuvant to chemotherapy for estrogen receptor-positive breast cancers like MCF-7. The cytotoxicity of the photodynamic therapy of MCF-7 breast cancer cells in a 3D cell culture with an ICG solution of 50 µM ICG concentration and 10^5^ cells/mL concentration is supported by the visual evidence provided with microscopic images also included in Supplementary Materials (Figure S1). The comparative morphological changes in the lysosomal, membrane and nuclear compartments of untreated control cells (Figure 9A,C,E) and PDT-treated cells (Figure 9B,D,F) emphasize a progressive disruption of morphological changes with increasing intensities in the ICG efficacy as a near-infrared photosensitizer to induce oxidative stress and structural collapse in estrogen receptor-positive models of breast cancer. The strong blue lysosomal staining of the control cells (Figure 9A) depicts a diffuse distribution of intact lysosomes in the cytoplasm, as expected given their involvement in cellular homeostasis by enzyme degradation and nutrient recycling. After PDT with ICG (Figure 9B), the virtual absence of this blue light is an indication that lysosomal membrane permeabilization (LMP) has occurred as one of the initial events of PDT-induced cell death. This permeabilization facilitates the release of the cathepsins and other hydrolytic enzymes into the cytosol, which further enhances the damage caused by the ROS and initiates downstream apoptotic or necrotic responses. Other LMPs have been reported in PDT regimens with phthalocyanine photosensitizers, with acridine orange quenching experiments showing that lysosomal destabilization is a major event, typically followed by mitochondrial destabilization. The role of LMP in therapy resistance modulation is specific to MCF-7 cells, since autophagy-deficient variants have an increased susceptibility to PDT because of the disruption of lysosomal repair mechanisms. These findings support the oxidative burst of the ICG photoactivation, which is concentrated in lysosomes, resulting in their destruction and the likely release of enzymes because of it, which enhances cytoplasmic proteolysis. The fact that the green FITC-based membrane staining also displays the loss of integrity in the control cells (Figure 9C), where a continuous green outline is a descriptive element of the intact cellular boundaries and spherical morphology of viable MCF-7 spheroids in 3D matrices. The lack of this signal in post-PDT therapy (Figure 9D) is an indication of extreme fragmentation and permeability of the plasma membrane, a direct result of lipid peroxidation due to the diffusion of ROS outside of lysosomes. This rupture aids the influx of extracellular dyes and ions, which signify secondary necrosis or late apoptosis, and is a dependable indication of inescapable cell death. The ICG translocation as a late-stage biomarker of PDT lethality is shown through nuclear assessment using Texas Red emission (Figure 9E,F). In unstimulated cells (Figure 9E), low fluorescence levels of red indicate impermeability of the nuclear envelope of the photosensitizer, which maintains genomic stability. By contrast, the vague, strong, red light observed in treated nuclei (Figure 9F) is indicative of envelope rupture, which permits ingress of ICG and condensation of chromatin, indicators of an apoptotic execution. The direct observation of ^1^O_2_ phosphorescence at 1270 nm in MCF-7 breast cancer cells incubated with a solution of 50 µM ICG and irradiated at 780 nm is a solid indication of highly effective phosphorescence. The symmetrical Gaussian shape, emission maximum at 1270 nm, full width at half maximum (FWHM) of about 750–800 nm, and the peak intensity of about ~0.38 a.u. are all in complete agreement with the typical NIR phosphorescence of ^1^O_2_ in aqueous and biological samples [61,62]. The high yield of ^1^O_2_ obtained at a solution of 50 µM ICG is highly comparable with earlier reports, which show that this concentration is in the range of optimum to facilitate the photodynamic action of ICG solution in different cell lines [63,64]. When the concentration is below 20 µg/mL, the quantity of internalized dye is not enough to generate ROS, and when it is above 100 µg/mL, the concentration of H-type aggregation and inner-filter effect is self-quenching, and quantum yield of singlet oxygen is reduced [26]. Numerous independent experiments using direct ^1^O_2_ phosphorescence measurements have found virtually the same spectral characteristics of ICG-sensitized systems, peak emission at 1268–1272 nm and lifetimes ranging between 3 and 5 µs in aqueous solution [65,66]. Further time-resolved, spectrally resolved measurements in bacterial biofilms indicated that signal intensity changes directly with ICG solution concentration (20–100 µg/mL) and directly with the dose of irradiated light, which is directly proportional to photocytotoxicity [67]. These data support the conclusion that the high ^1^O_2_ signal in the MCF-7 cells is genuine toxicity and not an artifact. Translationally, the capacity of ICG to produce elevated concentrations of ^1^O_2_ on near-infrared excitation (780–810 nm) is specifically beneficial to deep-tissue photodynamic therapy; the light at this wavelength has the highest tissue penetration and the lowest tissue absorption [68]. In addition, ICG has already been approved by the FDA as a clinical imaging agent; it has a high cellular uptake rate, which is facilitated by organic-anion-transporting polypeptides (OATPs) that are frequently overexpressed in breast cancer, and it is not toxic to cells, in the dark [69,70]. Together with the current firsthand evidence of strong ^1^O_2_ production in MCF-7 cells, these attributes make ICG a very promising imaging and therapy agent for breast cancer PDT. Overall, the given singlet-oxygen emission spectrum is clear evidence that ICG at a solution of 50 µM is an excellent near-infrared photosensitizer in MCF-7 breast cancer cells and has the capacity to produce cytotoxic ^1^O_2_ in large quantities, contributing significantly to the observed cytotoxicity, with great spectral selectivity. These findings are much in line with the accumulating evidence on direct ^1^O_2_ detection and go even further to endorse clinical investigation of ICG-based photodynamic therapy. In our study, the irradiation time was 12 min 34 s to achieve a sufficient fluence of 30 J/cm^2^ at a measured irradiance of 39.8 mW/cm^2^. A fluence of 30 J/cm^2^ is one of the most frequently reported values in studies of indocyanine green photodynamic therapy in breast cancer cell lines, including MCF-7, because it consistently induces strong cytotoxicity of typically 60–95% while maintaining adequate tissue oxygenation and minimizing photobleaching by ICG solution and excessive hyperthermia at low and medium irradiance [71,72,73]. The developed photodynamic procedure with ICG solution at a concentration of 50 µM and 780 nm light wavelength causes strong and statistically significant cytotoxicity against MCF-7 breast cancer cells (41.7% killed in 2D, 29.8% in 3D), with negligible toxicity towards healthy HMECs (only 9% reduction in viability, without statistical significance). These results indicate the high clinical potential of this method and justify further preclinical studies with parameter optimization in 3D and in vivo models. Other authors, using an alternative dye, 5-aminolevulinic acid (ALA), also demonstrated very low toxicity to healthy HMECs. The effect on HMECs remained minimal and did not significantly affect the number of viable cells in longer observations (up to 9 days). These results underscore the high selectivity of ALA-PDT, analogous to that observed in our study with ICG, where healthy cells showed only a ~9% reduction in viability after photothermal therapy [74]. The selective activity of ICG in PDT against breast cancer cells compared to normal epithelial cells is primarily due to greater ICG uptake in malignant cells, mediated by, among other factors, overexpression of the OATP1B3 transporter and higher endocytotic activity [75,76,77]. The mechanisms of ROS generation are similar in both cell types, but cancer cells are more sensitive due to weaker antioxidant defenses and higher ICG accumulation, leading to increased apoptosis and ferroptosis. Therefore, PDT therapy with ICG is a safe method of treating cancer while preserving healthy cells. The significantly lower cell kill rate of 29.8% in MCF-7 breast cancer cells embedded in 3D collagen, compared with the significantly higher efficacy in 2D monolayers, reflects a complex interplay of physiological barriers that mimic the in vivo tumor microenvironment more closely than flat cultures. These barriers extend far beyond simple limitations of light or ICG penetration to include hypoxic gradients, ECM-dependent resistance mechanisms, and physicochemical challenges specific to photosensitizers. Reduced oxygen diffusion and increased oxygen consumption in the dense 3D collagen matrix are considered the main limiting factors, leading to the rapid development of hypoxic areas, especially in the spheroid core. The efficacy of PDT therapy is critically dependent on molecular oxygen for the generation of cytotoxic singlet oxygen and other ROS. Hypoxia, therefore, significantly impairs ROS production and cell killing. This phenomenon is consistently observed in three-dimensional MCF-7 spheroid models, where deeper light penetration and dosimetry correction are required to overcome oxygen limitations [78,79,80]. Furthermore, ECM interactions in three-dimensional cultures induce phenotypic changes, including increased cell adhesion to the ECM, enhanced expression of survival signaling pathways, and multicellular resistance mechanisms that collectively desensitize cells to phototherapy-induced apoptosis. Such ECM-dependent resistance has been shown to modulate therapeutic response in various three-dimensional systems, with invasive ECM conditions sometimes paradoxically altering susceptibility but generally providing greater overall protection compared to two-dimensional systems [81,82]. Finally, ICG accumulation in a confined 3D environment can promote molecular aggregation, leading to aggregation-induced quenching (ACQ) and self-quenching effects. This reduces both fluorescence emission and the singlet-oxygen quantum yield, thereby reducing photodynamic activity even in the presence of sufficient light and oxygen. This issue is particularly relevant for ICG-based PDT, as aggregation under physiological or matrix-rich conditions is a well-documented limitation [83,84].

### 3.1. Limitations Between 2D and 3D Cell Models

In 2D monolayers, PS molecules have direct, uniform access to cells, leading to higher uptake and intracellular accumulation. This often results in greater ROS production and cytotoxicity. In 3D models, diffusion barriers from ECM components (e.g., collagen) and cell packing limit PS penetration, creating concentration gradients where outer layers absorb more PS than inner cores [85]. Light scattering and absorption are minimal in 2D, allowing uniform illumination. In 3D, tissue-like density reduces fluence (light energy delivery) to deeper layers, in spheroids larger than 200–300 μm, leading to suboptimal activation in cores [86]. Studies using LED sources at various wavelengths (e.g., 635–810 nm) in cervical carcinoma cells demonstrated that 3D cultures require higher light doses for equivalent cytotoxicity, with 2D models showing up to twice the sensitivity [87]. PDT relies on normoxic conditions for type II mechanisms. 2D cultures are typically well-oxygenated, promoting efficient ROS generation [88]. 3D models develop hypoxic gradients, shifting PDT toward less efficient type I mechanisms and increasing resistance. Hypoxic 2D cultures sometimes mimic 3D normoxic responses, suggesting oxygen as a key modulator [89]. 2D models often overestimate apoptosis due to uniform exposure, while 3D introduces heterogeneity, with peripheral cells dying via apoptosis and central cells via necrosis or surviving due to hypoxia [90]. Across studies, 3D models consistently show lower PDT efficacy and less cell kill than 2D under identical conditions, due to the combined effects above [91]. This “mind the gap” phenomenon underscores 3D’s value in predicting in vivo challenges, such as tumor architecture and stromal interactions, while supporting combination therapies. In the provided study, ICG-PDT in MCF-7 cells aligns with these patterns. 2D monolayers showed higher efficacy (41.7% cell kill, viability 58.3%) than 3D collagen matrices (29.8% kill, viability 70.2%), with statistical significance (*p* = 0.047 between models). This ~1.4-fold difference reflects reduced ICG uptake (78% in 2D vs. 65% in 3D) and linear depth-dependent penetration in 3D, mirroring the literature on diffusion barriers in hydrogels. The study also confirms singlet oxygen as the primary cytotoxic species via 1270 nm phosphorescence, but the lower 3D efficacy highlights hypoxia and ECM limitations, consistent with spheroid studies, where core resistance persists. Selectivity against normal HMECs (91% viability) is preserved across models, suggesting ICG’s potential as a theragnostic agent, but 3D data better predicts clinical challenges in solid tumors. Future studies could incorporate co-cultures or advanced imaging to further bridge these gaps.

### 3.2. Comparison of ICG with Other Photosensitizers Used in PDT of Breast Cancer

PDT has emerged as a promising minimally invasive approach for breast cancer treatment, utilizing a wide spectrum of photosensitizers that differ substantially in photophysical properties, cellular uptake, intracellular localization, and mechanisms of cell death induction. Among the most extensively studied photosensitizers are porphyrins, chlorins, phthalocyanines, and 5-aminolevulinic acid (5-ALA)-derived protoporphyrin IX (PpIX), each demonstrating variable efficacy in breast cancer models [92,93]. While these agents often achieve pronounced cytotoxicity in vitro, their clinical translation is frequently limited by prolonged photosensitivity, insufficient tissue penetration, or lack of selectivity toward malignant cells. Porphyrin- and phthalocyanine-based photosensitizers have demonstrated strong phototoxic effects in MCF-7 and MDA-MB-231 breast cancer cell lines, primarily through efficient generation of ROS and induction of apoptosis [94,95,96]. Gallium and zinc phthalocyanine derivatives, for example, were shown to significantly reduce MCF-7 cell viability following red-light activation, often achieving greater than 50% cell death under optimized conditions [95,96]. However, repeated PDT cycles with these sensitizers have been reported to induce partial resistance in MCF-7 cells, highlighting a potential limitation associated with their long intracellular retention and adaptive cellular responses [97]. 5-ALA-based PDT remains one of the most widely investigated approaches in breast cancer due to its endogenous conversion into PpIX. Studies have demonstrated dose- and light-dependent cytotoxicity in both hormone-positive and triple-negative breast cancer cell lines [98,99,100]. Nevertheless, the efficacy of 5-ALA-PDT is highly dependent on metabolic activity and heme synthesis pathways, which can vary significantly between tumor subtypes and microenvironmental conditions, particularly in 3D culture systems and hypoxic tumor cores. To overcome limitations of free photosensitizers, numerous studies have explored nanoparticle-based delivery systems incorporating chlorins, phthalocyanines, or ICG. These strategies frequently enhance intracellular accumulation and PDT efficacy in breast cancer models, including MCF-7 and 4T1 tumors, and may exhibit synergistic effects when combined with chemotherapeutic agents such as doxorubicin or paclitaxel [97,101]. However, increased formulation complexity, regulatory hurdles, and potential long-term toxicity of nanocarriers remain challenges for clinical translation. In this context, ICG represents a distinct class of photosensitizer with several translational advantages. As an FDA-approved near-infrared dye, ICG offers deep-tissue penetration, rapid systemic clearance, and an excellent safety profile. Previous studies have demonstrated that ICG-mediated PDT induces ROS generation and cytotoxicity in breast cancer cells, with enhanced selectivity toward malignant cells compared to normal mammary epithelial cells [102]. Importantly, ICG-PDT has been shown to be effective in both 2D and advanced 3D breast cancer models, although reduced efficacy in 3D systems reflects diffusion and oxygen limitations characteristic of solid tumors. Compared with traditional photosensitizers, the present findings support ICG as a highly attractive theragnostic agent, combining imaging capability with selective photodynamic cytotoxicity. The direct spectroscopic detection of singlet-oxygen generation presented here provides unequivocal mechanistic confirmation of photodynamic action, which is rarely demonstrated in comparable breast cancer PDT studies. When benchmarked against porphyrins, phthalocyanines, and 5-ALA-based systems, ICG demonstrates competitive cytotoxic efficacy while offering superior clinical readiness and safety. Collectively, these comparisons highlight the potential of ICG-based PDT as a simplified, clinically translatable strategy for selective photodynamic treatment of breast cancer and justify further preclinical and in vivo investigations.

## 4. Materials and Methods

### 4.1. General Information

Indocyanine green was manufactured by Carl Roth (Karlsruhe, Germany). CytoPainter lysosomal blue stain and Live/Dead cell assay were purchased from Abcam Inc. (Cambridge, MA, USA). Mitotracker green stain was supplied by Sigma Aldrich (Warsaw, Poland). Fetal Bovine Serum (FBS, 3D cell culture validated) was purchased from Sigma Aldrich (Warsaw, Poland). RPMI 1640 media and penicillin/streptomycin were purchased from Sigma Aldrich (Warsaw, Poland). Phosphate-buffered saline (PBS) was purchased from Sigma Aldrich (Warsaw, Poland). Collagen (Type I, 95% powder), Trypan Blue, and DMSO were purchased from Sigma Aldrich (Warsaw, Poland). Microscopic glass slides (sized 100 mm × 30 mm and thickness 0.96 ± 0.1 mm) were purchased from Sigma Aldrich (Warsaw, Poland). The hemocytometer used was from Hausser Scientific (Horsham, PA, USA). Round-bottomed, ultra-low-attachment 24-well plates (Corning^®^ Costar^®^ Ultra-Low Attachment, cat. No. CLS3473) were purchased from Corning Inc. (Corning, NY, USA). Sterile 3 mL vials were purchased from Sigma Aldrich (Warsaw, Poland). We used AquaB Duo reverse osmosis system manufactured by Fresenius Medical Care in Singapore. UV-VIS spectra were collected on an Agilent 8453 spectrophotometer (Warsaw, Poland). Images of cells were obtained with a microscope Zeiss Axio Imager.D2 (Oberkochen, Germany) equipped with 10×, 20× and 100× objectives (Zeiss Plan-Apochromat) and Zeiss Primovert (Oberkochen, Germany). All probes were pinned to a precision mechanical XYZ translation stage (Thorlabs, Newton, NJ, USA) that provides precise, controlled linear movement along the X, Y, and Z axes with sub-millimeter accuracy

### 4.2. Cell Culture Models

All cell culture procedures for MCF-7 and HMECs were performed in a sterile Class II biological safety cabinet Bio Hood 2 (MICROZONE, Ottawa, ON, Canada). For all tumor and normal breast cell types, in vitro culture conditions in culture medium were applied. The MCF-7 cells (ATCC^®^, Manassas, VA, USA) were cultured in RPMI medium (Biofluids) containing 5% fetal calf serum (Biofluids), 2 mM glutamine, 50 units/mL penicillin, and 50 µg/mL streptomycin. HMEC from ATCC^®^ (Manassas, VA, USA) were isolated from mammoplastic tissue and cultured in flasks until reaching 10^5^ cells/mL in Medium 171 supplemented with 0.4% bovine extract, 5 mg/L bovine insulin, 0.5 mg/L hydrocortisone, and 3 µg/L human epidermal growth factor.

#### 4.2.1. The 2D Cell Culture

The suspension of 10^6^ cells was incubated at 37 °C in a humidified incubator with 5% CO_2_. After 24 h, the medium was replaced and subsequently changed 2–3 times per week according to ATCC recommendations. Once the culture reached approximately 90% confluence, the medium was discarded, and 3 mL of 0.05% trypsin-EDTA (Sigma-Aldrich, Burlington, MA, USA) was added to the flask. The cells were incubated for 3–5 min at 37 °C in the CO_2_ incubator until detachment was observed under the microscope. The cell suspension was collected into 15 mL conical tubes, and the culture flask was rinsed with PBS to collect any remaining cells. The suspension was then centrifuged at 500× *g* for 5 min. Following cell counting, cells were seeded in 24-well plates at a final concentration of 1 × 10^5^ cells per well in 3 mL of complete medium. Plates were incubated at 37 °C in a 5% CO_2_ atmosphere. The cells adhered and spread on the plastic surface.

#### 4.2.2. 3-Dimensional Cell Cultures

The 3D model employed here is a hybrid collagen sandwich system, where MCF-7 cells are embedded as a layer between two polymerized type I collagen matrices on a microscopic slide, mimicking aspects of the tumor extracellular matrix (ECM) without forming true spheroids or incorporating perfusion**.** We used ultra-low-attachment, round-bottomed 24-well plates (Corning^®^ Costar^®^) that, in contrast to standard flat-bottom plates, do not require coating to prevent cell adhesion. The well shape promotes the formation of single, centrally located spheroids of reproducible size. Optimal seeding densities were established for each cell line. 3D cell cultures were prepared with 10^5^ MCF-7 cells. Cells were counted to the appropriate number and added to 400 µL culture media to achieve a final concentration of 10^5^ cells/400 µL. Cells were then incubated for 30 min in 5% CO_2_ at 37 °C. A collagen matrix was prepared on ice at a concentration of 1.6 mg/mL by neutralizing an acidic collagen solution. To neutralize collagen, 100 mM of HEPES buffer was used. The buffer was first prepared in a 2× PBS solution and mixed with the same amount of acidic collagen solution (1:1 ratio). The collagen concentration of 1.6 mg/mL was adjusted by adding culture media. Preparation of collagen for 3D matrices was done in about 60 min. Collagen can be stored on ice for only one hour; therefore, the cells were prepared immediately. The microscopic slide was placed on ice. Cell culture media was warmed to 37 °C on water bath. Then, 15 µL of ice-cold collagen Type 1 solution was placed on the surface of the slide using an Eppendorf pipette. The 15 µL collagen solution was gently spread on the microscopic slide using the pipette tip. The slide was incubated at 37 °C and 5% CO_2_ in the incubator for 25 min to allow collagen to polymerize. By warming the collagen to 37 °C, the polymerization process produces the fibrillar matrix. At this time, the matrix was easily detached from the slide. The polymerization of the matrix must be efficient to avoid the cell suspension adhering to the surface only or the floating of the matrices. The collagen matrix was drying over time but was not completely dried, to avoid changes in the physical properties of the matrix. During the time of collagen polymerization, the cell suspension was prepared. The microscopic slide was removed from the incubator and placed on Kimberly Clark paper tissue under the hood to avoid a lower temperature that could destabilize the collagen matrix. Then, 400 µL of media with 10^5^ MCF-7 cells in suspension was added to the top of polymerized collagen. The microscopic slide with a collagen matrix that was incrementally populated with the cells was incubated at 37 °C and 5% CO_2_ in a cell culture incubator for 30 min. The incubation was necessary to allow cells to attach to the collagen matrix. A microscope equipped with a 10× objective was used to check if cells attached to the matrix. The microscopic slide was placed on the microscope stage and gently shaken. The cells attached to the collagen matrix did not move separately or with the media. Cells were embedded in collagen. At this time, non-attached media without cells (~10 µL of media that did not attach to the collagen) was gently removed from the slide using a 20 µL Eppendorf pipette. In the next step, a ~80 µL drop of collagen was applied on top of the cells in collagen gel to adhere to the first collagen layer. The slide was placed into the 37 °C and 5% CO_2_ incubator and incubated for 30 min to polymerize the second layer of fibrillar collagen. The collagen layer was again confirmed with the microscope using 10× objective. In the last step, about 100 µL of cell culture media was pre-warmed to 37 °C and added to the well at room temperature. The formed 3D setup was again placed into the 37 °C incubator and incubated for 24 h to allow cells to establish polarity and start migrating in the 3D cell cultures.

### 4.3. Cell Viability

After centrifugation, the cell pellet was resuspended in complete medium. A 20 μL aliquot of the cell suspension was transferred into an Eppendorf tube, and 380 μL of Muse^®^ Cell Count & Viability reagent (Luminex, Austin, TX, USA) was added. The mixture was incubated at room temperature for 5 min. Cells were then counted using the Guava^®^ MUSE^®^ Cell Analyzer (Cytek Biosciences B.V., Amsterdam, The Netherlands).

### 4.4. IC_50_ Measurements

Cells were seeded in 24-well plates at 1 × 10^5^ cells/mL. After 24 h, medium was replaced with 100 µL of fresh medium containing ICG at final concentrations of 0, 1, 5, 10, 25, 50, 75, 100, 150, 200, 250, and 300 µM. A 5 mM ICG stock was prepared in 2% DMSO/H_2_O and diluted in culture medium (final DMSO ≤ 0.1%). Following 2 h incubation with ICG, cells were washed twice with ice-cold PBS, irradiated with a 780 nm laser and cultured in complete medium for 24, 48, or 72 h. Cell viability was assessed using the Muse^®^ Cell Analyzer with the Muse^®^ Count & Viability Kit according to the manufacturer’s protocol. Cells were trypsinized (0.05%, 3 min), resuspended in 100 µL PBS, and analyzed. Results were expressed as the percentage of viable cells. Inhibitory concentration 50% (IC_50_) values were determined by nonlinear regression using GraphPad Prism 9.0 (*n* = 3 biological replicates, 3 technical replicates each.

### 4.5. Subcellular Localization of Sensitizer

The subcellular localization of ICG in a film of 10^5^ MCF-7 cells on a glass slide was performed. The cells were seeded 24 h prior to the experiment and incubated with a solution of 50 µM of ICG (dissolved in DMEM supplemented with 10% FBS) in the dark at 37 °C for 30 min. MitoTracker Green FM or CytoPainter Lysosomal Staining Blue was added into the media at a final concentration of 200 nM and 200 nM, respectively, during the last 15 min of ICG incubation. At the end of 30 min incubation, the cells were washed three times with PBS (pH 7.4). The fluorescence of MitoTracker Green (excitation/emission, 490/520 nm), CytoPainter Lysosomal Blue (excitation/emission, 350/440 nm), and ICG (excitation/emission, 780/820 nm) was detected with an epifluorescence microscope. The fluorescent images were acquired with confocal software using a 20× objective lens. The lysosomal blue dye permeates cells and accumulates in lysosomes via a pH gradient in amounts ranging from 10^15^ to 10^20^ mol/cell. ICG exhibited punctate perinuclear fluorescence that strongly colocalized with CytoPainter Lysosomal Blue (Pearson’s coefficient > 0.85), indicating predominant lysosomal localization, whereas no significant overlap was observed with MitoTracker Green (Pearson’s coefficient < 0.25), confirming the absence of mitochondrial accumulation in MCF-7 cells under these conditions.

### 4.6. Evaluation of ICG Uptake in 2D and 3D Cell Cultures

2D cell and 3D cell cultures were incubated with solutions of 50 µM ICG in the dark for 30 min. The uptake of ICG was acquired by flow cytometry Guava^®^ MUSE^®^ Cell Analyzer (Cytek Biosciences B.V., Amsterdam, The Netherlands), and 10^4^ cells per sample were analyzed in 3 independent experiments [103].

### 4.7. Penetration of ICG in 3D Cell Cultures

The penetration of ICG in 3D cell cultures was assessed using a Leica TCS SP2 confocal laser-scanning microscope (Leica Microsystems, Wetzlar, Germany). After 30 min incubation with 50 µM ICG in the dark, Z-stacks were acquired through the entire depth of the collagen matrix with a 5 µm step size using a 20× objective. Excitation was performed at 780 nm, and emission was collected between 800 and 850 nm (peak at 820 nm). Images were processed with Leica LAS AF Lite 4.0 software. Penetration depth was quantified as the distance from the surface where fluorescence intensity dropped to 50% of the maximum.

### 4.8. Photocytotoxicity Assay

**Figure 11:**
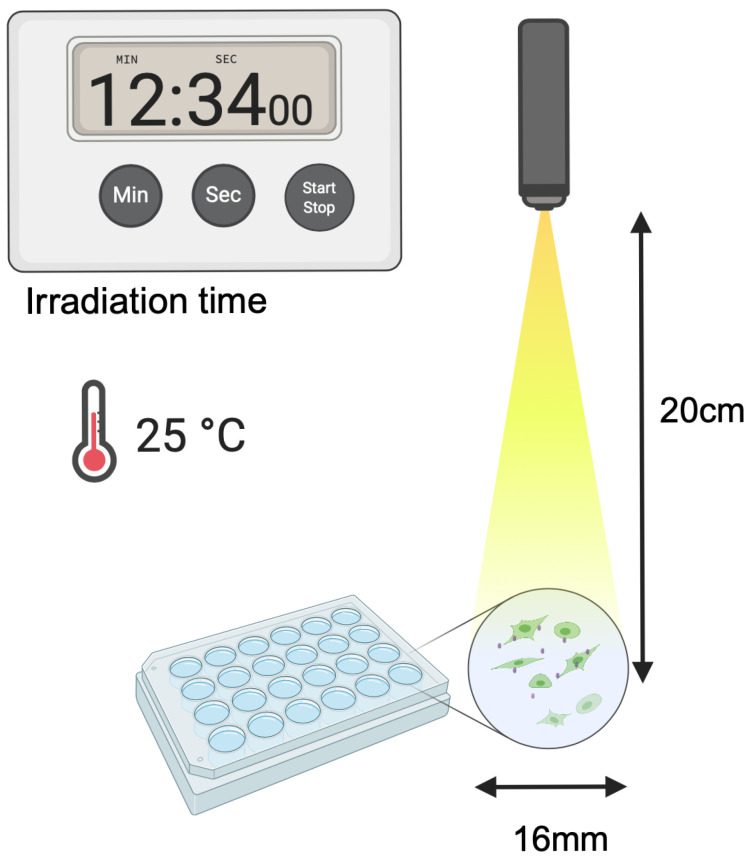
Experimental setup ensuring identical light dose delivery in 2D and 3D photodynamic therapy with ICG. The cell exposure time was 12 min and 34 s. The laser distance from the cells was 20 cm, and the surface diameter was 16 mm. Created with Created in BioRender. Mytych, W. (2026) https://BioRender.com/ygfx9ik (accessed on 29 January 2026).

The cells were washed three times with PBS, and fresh complete medium was added. Both 2D and 3D cell cultures were irradiated under identical geometric conditions to ensure comparable light dose delivery (Figure 11). Irradiation was performed for 12 min 34 s at room temperature (25 °C) using a VisIR-780 picosecond-pulsed laser (PicoQuant GmbH, Berlin, Germany) operating at 780 nm. The laser output power was adjusted to ~250 mW (average power). The beam was delivered via a single-mode fiber, terminated by an adjustable achromatic collimator, and defocused/homogenized using a ground-glass diffuser (Thorlabs, Newton, NJ, USA) placed immediately above the culture plate to ensure uniform conical illumination over the entire multiwell-plate area. The distance from the diffuser/collimator output to the cell monolayer was fixed at 20 cm. The irradiance at the cell plane was measured as approximately 40 mW/cm^2^ (using an optical power meter, averaged over the illuminated area). Fluence was calculated as irradiance × time, resulting in 30 J/cm^2^. This setup provided uniform exposure regardless of culture dimensionality due to the shared point-source-like geometry and perpendicular sample orientation.

In 3D the subcellular localization of ICG in a film of MCF-7 cells on a glass slide. ICG was added to the media at a final concentration of 50 µM. The cells were then incubated in the dark at 37 °C for 30 min. At the end of 30 min incubation, the cells were washed with PBS. The fluorescence of indocyanine green (excitation/emission, 780/820 nm) was detected with a fluorescence microscope. The fluorescent images were acquired with confocal software using a 10× objective lens. After treatment, cells in 2D and 3D collagen gel were stained with Live/Dead dye. The dye provided in the Live/Dead assay was diluted in PBS 5-fold, and 20 µL of it was used promptly after dilution for each sample of adherent cells.

### 4.9. Fluorescence Assay

Fluorescence imaging with a resolution of 2.0 × 2.7 microns with an objective of 10× was used to track the sensitizer and assess the concentration. The fluorescence intensity was correlated to the concentration of ICG, although aggregation phenomena for reduced fluorescence were not scrutinized. The approximate depth of cell film was 50–100 µm, which roughly matches the fluorescence imaging depth. These values are similar to the optical properties of breast tissue, which possesses an optical diffusion in the range of 40 to 100 µm [104,105,106]. Using an Eppendorf pipette, a 1 mL volume of 10^5^ cells was transferred to the surface of the microscopic glass slide and placed on an area of 625 mm^2^. The cells appeared to adhere to the microscope slide. We scanned the slide to find the different cellular morphologies in the ^1^O_2_ device-treated sample. We increased the magnification by switching to the next-highest-powered objective, 100×, and again scanned the slide. Using a total magnification of 10×, we observed the increase in the size of the image and the cellular detail at the higher magnification; the distance between cells fit with the media. The entire surface, 625 mm^2^, was monitored under a microscope, and 6 pictures were saved from one microscopic slide. For the pictures, an area from the center of the slide was selected.

### 4.10. Phosphorescence

For phosphorescence spectra we used a fully automated fluorescence spectrometer FluoTime 300 “EasyTau” (PicoQuant, Berlin, Germany), equipped with options for phosphorescence measurements, including singlet oxygen emission in the NIR range. We used a picosecond-pulsed diode laser as the ICG excitation source. The emission was collected at an angle of 90° using an NIR-sensitive detector with a monochromator. The EasyTau 2 software automated the setup, i.e., wavelength selection, slit adjustment (10 μm–4 mm), spectrum acquisition, and kinetics. The data was analyzed in FluoFit with exponential fitting and quality assessment. To optimize sensitivity and minimize noise, the PMT detector was thermoelectrically cooled to reduce dark counts. We used long-pass filters (scattered light rejection 1:10^5^–1:10^10^) and overload protection. The monochromator was set to 1270 nm with a resolution of ≤0.1 nm and with NIR-optimized gratings. The sample was placed in a 1 cm cuvette in a multifunctional chamber at a temperature of 15 °C. The entire setup was controlled by software—there were no manual adjustments of the optics during measurement.

### 4.11. Statistical Analysis

Results are expressed as the mean ± S.D. The statistical significance of differences between the groups was determined by applying an ANOVA (Analysis of Variance); one-tailed test with Welch’s correction; Student’s *t*-test; Durbin–Watson statistic and Shapiro–Wilk test. Values of *p* < 0.05 were considered statistically significant.

## 5. Conclusions

This study demonstrates that ICG, at a clinically achievable concentration of 50 µM solution and activated by 780 nm NIR light, functions as an effective and selective photosensitizer for photodynamic therapy of MCF-7 breast cancer cells. In 2D monolayers, ICG in photodynamic therapy achieved 41.7% tumor cell kill (viability 58.3 ± 7.4%, *p* < 0.0001), whereas in more physiologically relevant 3D cell cultures, the kill rate was 29.8% (viability 70.2 ± 10.7%, *p* = 0.0002), reflecting realistic limitations of dye and light penetration seen in solid tumors. Importantly, normal human mammary epithelial cells retained 91.0 ± 1.3% viability under identical treatment conditions (therapeutic index ≈ 4.6), confirming high cancer cell selectivity. Direct time-resolved NIR measurement provided unambiguous evidence of singlet-oxygen generation (^1^O_2_ phosphorescence peak at 1270 nm, intensity~0.38 a.u.). The observed time-dependent decrease in IC_50_ (from 51.4 µM at 24 h to 27.3 µM at 72 h) and rapid loss of lysosomal and membrane integrity further support progressive oxidative damage leading to cell death. These findings, combined with ICG’s established safety profile, rapid clearance, and existing regulatory approval for human use, position ICG as a promising photosensitizer for PDT, a low-cost theragnostic approach for breast cancer that simultaneously enables real-time fluorescence-guided treatment. Future optimization of delivery strategies and validation in orthotopic and patient-derived xenograft models are warranted to translate this simple yet powerful modality into clinical practice.

## Figures and Tables

**Table 1:** Average results obtained from MCF-7 breast cancer cell viability measurements using 2D and 3D cultures vs. results obtained from 2D HMEC culture.

Setup/Cell Culture	HMEC	MCF-7	
	2D	2D	3D
Control	98.1 ± 0.1%	97.5 ± 0.6%	96.4 ± 0.9%
Cells with ICG without light irradiation	96.5 ± 0.8%	94.92 ± 0.2%	91.17 ± 2.93%
Cells without ICG irradiated with 780 nm light	96.7 ± 0.91%	89.7 ± 1.2%	90.83 ± 3.93%
Cells with solution of 50 µM ICG irradiated with 780 nm light	90.98 ± 1.3%	58.34 ± 7.4%	70.17 ± 10.65%

## Data Availability

All data has been included.

## Supplementary Materials

The following supporting information can be downloaded at: https://www.mdpi.com/article/10.3390/molecules31030520/s1, Figure S1: Microscopic images of single cancer cell. (A) black and weight microscopic image of live cell with a diameter of 20 microns before photocleaving experiments (B) fluorescence image of dead cell showing ICG sensitizer uptake (red color emission (C) live cells with lysosomal staining and (D) live cells with green staining of cellular membrane. (A) shows a bright field (black and white) image of a live cell, revealing overall cell morphology with visible nucleus cytoplasm contrast and no fluorescence, serving as a structural reference. (B)displays a fluorescence image of a dead cell with intense red emission, indicating ICG uptake and retention throughout the cell volume; this dye is excited in the near-infrared range and emits in the red spectrum with the diffuse signal distribution suggesting presence in the cytoplasm and possibly organelles. (C) depicts a live cell with blue lysosomal fluorescence, where lysosomes appear as punctate structures mainly in the perinuclear region, without significant colocalization with ICG solution. (D) shows a live cell with green fluorescence of the cell membrane, highlighting a continuous and smooth plasma membrane contour, confirming cell integrity and viability. Comparison of live (A,C,D) and dead (B) cell images reveals loss of compartmentalization upon death, with diffuse ICG distribution. Overall, ICG is internalized by the cancer cell, localizes primarily in the cytoplasm, shows no strong colocalization with lysosomes or the cell membrane, and its retention after cell death enables tracking of therapy efficacy. Suggested further studies include colocalization analyses with markers of other organelles, time-lapse observations of uptake in live cells, and tests of NIR irradiation response to assess reactive oxygen species generation.
