# Supplementary material for: Indocyanine Green as a Theragnostic Agent in MCF-7 Breast Cancer Cells
Source: Molecules. 2026 Feb 2;31(3):520. doi: 10.3390/molecules31030520 (PMC12899179; doi:10.3390/molecules31030520)
Supplement: Supplementary file 1 [file molecules-31-00520-s001.zip › molecules-4103949-supplementary.pdf]

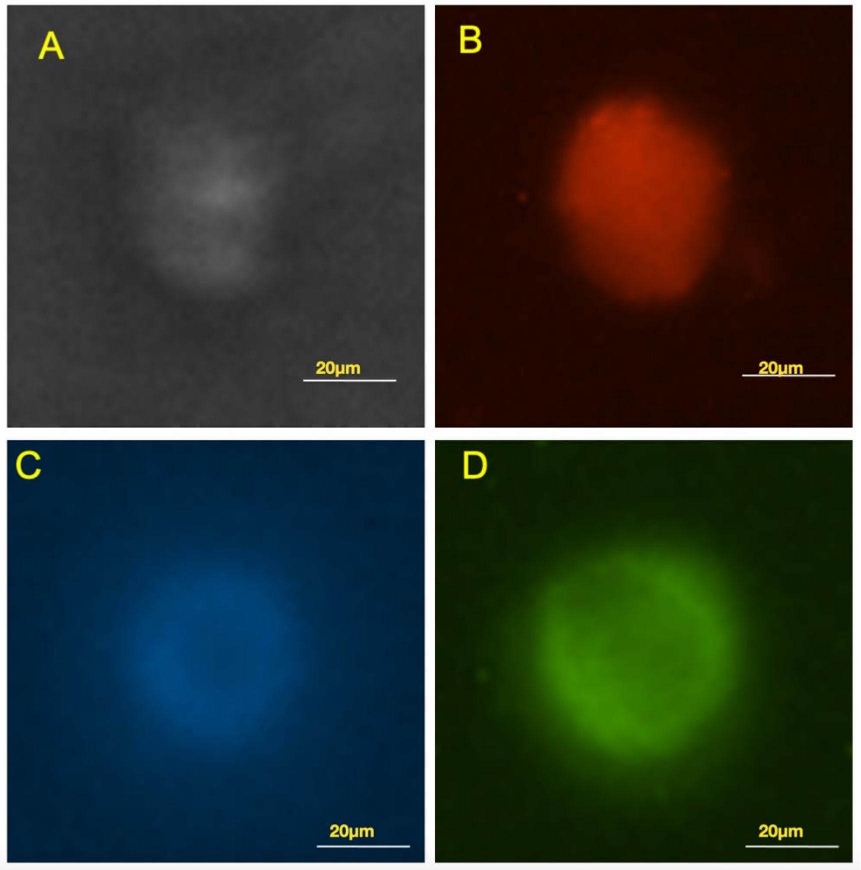

Figure S1. Microscopic images of single cancer cell. (a) black and weight microscopic image of live cell with a diameter of 20 microns before photocleaving experiments (b) fluorescence image of dead cell showing ICG sensitizer uptake (red color emission (c) live cells with lysosomal staining and (d) live cells with green staining of cellular membrane. Panel A shows a bright field (black and white) image of a live cell, revealing overall cell morphology with visible nucleus cytoplasm contrast and no fluorescence, serving as a structural reference. Panel B displays a fluorescence image of a dead cell with intense red emission, indicating ICG uptake and retention throughout the cell volume; this dye is excited in the near-infrared range and emits in the red spectrum with the diffuse signal distribution suggesting presence in the cytoplasm and possibly organelles. Panel C depicts a live cell with blue lysosomal fluorescence, where lysosomes appear as punctate structures mainly in the perinuclear region, without significant colocalization with ICG solution. Panel D shows a live cell with green fluorescence of the cell membrane, highlighting a continuous and smooth plasma membrane contour, confirming cell integrity and viability. Comparison of live (A, C, D) and dead (B) cell images reveals loss of compartmentalization upon death, with diffuse ICG distribution. Overall, ICG is internalized by the cancer cell, localizes primarily in the cytoplasm, shows no strong colocalization with lysosomes or the cell membrane, and its retention after cell death enables tracking of therapy efficacy. Suggested further studies include colocalization analyses with markers of other organelles, time-lapse observations of uptake in live cells, and tests of NIR irradiation response to assess reactive oxygen species generation.
